# The glycosylation variant at residue 381 of the spike protein contributes to virulence shifts in porcine epidemic diarrhea virus during both natural field transmission and laboratory cell passaging with poor cross-protection

**DOI:** 10.1128/jvi.01561-25

**Published:** 2025-11-24

**Authors:** Zhiwei Li, Zhiqian Ma, Yongqi Li, Xiaojing Zhao, Yonghui Zheng, Yang Li, Yingtong Feng, Xuyang Guo, Zifang Zheng, Lele Xu, Jianwu Zhang, Haixue Zheng, Shuqi Xiao

**Affiliations:** 1 State Key Laboratory for Animal Disease Control and Prevention, College of Veterinary Medicine, Lanzhou University, Lanzhou Veterinary Research Institute, Chinese Academy of Agricultural Sciences 111658 , Lanzhou, Gansu, China; 2 Gansu Province Research Center for Basic Disciplines of Pathogen Biology, Lanzhou Veterinary Research Institute, Chinese Academy of Agricultural Sciences 111658 , Lanzhou, Gansu, China; University of Michigan Medical School, Ann Arbor, Michigan, USA

**Keywords:** porcine epidemic diarrhea virus, glycosylation mutation, viral pathogenesis, cross-protection, spike protein

## Abstract

The virulence and immunogenicity of porcine epidemic diarrhea virus (PEDV) vary during field circulation and cell culture passage (such as when the GI CV777 strain is attenuated through serial passaging). This study revealed that the glycosylation site mutation at position 381 (N381K) of the S protein is associated with these phenomena. Compared with piglets inoculated with P13 virus, piglets inoculated with P100 (N381K) of virulent GX223 exhibited delayed diarrhea, viral shedding, and mortality. Using the virulent rCH/SX/2016-S_HNXP_ strain (rPEDV-S_wt_) as the backbone, we generated rPEDV-S_N381K_. While both the wild-type and mutant strains showed similar growth *in vitro* and in 2-day-old piglets, rPEDV-S_N381K_ caused milder diarrhea and lower mortality. In 5-day-old piglets, the mutant strain also induced delayed viral shedding and milder diarrhea. At 21 days after post-infection, all the piglets were challenged with the parental strain. The pigs in both the rPEDV-S_N381K_ and rPEDV-S_wt_ groups produced high IgA/IgG levels, but the rPEDV-S_N381K_-inoculated piglets presented higher fecal viral loads and lower neutralizing antibody titers against the parental strain. Molecular modeling suggests that N381K alters antigenic epitope interactions, which may affect virulence and immunogenicity. While this study has the limitation of a relatively small sample size in the animal studies, the results collectively demonstrate that S protein glycosylation mutations influence PEDV virulence and contribute to reduced cross-protective vaccine efficacy, offering important insights for PEDV pathogenesis research and vaccine development.

**IMPORTANCE:**

Porcine epidemic diarrhea virus (PEDV) continues to cause substantial economic losses in the global swine industry, with emerging strains challenging existing vaccine strategies. This study identifies the N381K glycosylation site mutation in the S protein of PEDV as a factor involved in variations in virulence during natural transmission and laboratory adaptation. Crucially, the mutant induces suboptimal neutralizing immunity against the prevalent strain, revealing a mechanism by which classical-strain vaccines may provide limited protection against currently circulating strains. Our findings reveal how a single glycan modification modulates both pathogenicity and immunogenicity, providing critical insights for the development of effective vaccines against circulating PEDV variants.

## INTRODUCTION

Porcine epidemic diarrhea virus (PEDV) is a single-stranded positive-sense RNA virus of the genus Alphacoronavirus (1). The approximately 28-kb viral genome consists of the 5′ untranslated region (5′ UTR), open reading frame 1a/1b (ORF1a/1b) encoding replicase polyproteins pp1a and pp1ab, the spike (S) gene, the accessory protein-encoding gene (ORF3), the envelope (E) gene, the membrane (M) gene, the nucleocapsid (N) gene, the 3′ untranslated region (3′ UTR), and the poly(A) tail (2). PEDV has evolved into two major genogroups: GI (classical) and GII (variant). These genogroups further diversified into subgroups: GI split into GI-a and GI-b, while GII subdivided into GII-a, GII-b, and GII-c (3). The GI-a subgroup predominantly comprised early European strains, including the virulent CV777 and DR13 prototypes. In contrast, GI-b contains cell culture-adapted vaccine strains (attenuated CV777 and DR13) along with pandemic classical strains circulating in Asia, and most GI-b PEDV strains are vaccine strains or vaccine-like strains with attenuated virulence (3, 4).

Coronaviruses, as RNA viruses, exhibit high genomic variability, which significantly influences their transmissibility, virulence, and immunogenicity (5–8). PEDV was first identified in Belgium and the United Kingdom in 1976, with subsequent reports emerging from China during the 1980s, where it exhibited a sporadic and geographically limited distribution (9, 10). However, severe epidemics occurred in 2010, when highly virulent variant PEDV strains emerged in China (11). These highly virulent variants rapidly disseminated globally, resulting in near-catastrophic impacts on the swine industry, but the mechanism underlying the enhanced virulence of the variant strains remains unclear. Live attenuated vaccines (LAVs) are widely adopted for disease prevention because of their superior immunogenicity and protective efficacy (12). Commercial PEDV LAVs (such as those from the CV777 and DR13 strains) are typically generated through serial passaging in nonporcine cell lines (such as VERO cells), although the molecular mechanisms underlying their attenuation remain poorly characterized. However, the efficacy of classical-strain vaccines against currently circulating epidemic strains has been proven inadequate (13). The mechanisms responsible for this limited cross-protection remain to be elucidated.

The S protein of coronaviruses is a highly glycosylated multifunctional protein that plays critical roles in viral entry, replication, virulence, and tropism (14–17). Deletion of both N331 and N343 glycosylation of severe acute respiratory syndrome coronavirus 2 (SARS-CoV-2) S protein reduced viral infectivity. The N234Q and N165Q mutations altered viral sensitivity to neutralizing antibodies (18). Mutations in the N-terminal domain (NTD) of the SARS-CoV-2 XEC variant affect immune evasion, cell–cell fusion, and S protein stability (19). However, whether glycosylation mutations in the PEDV S protein contribute to variations in viral virulence remains to be elucidated.

In this study, the N381K glycosylation mutation in the PEDV S protein, which was identified in both cell culture-attenuated GI vaccine strains, low-virulence GI-b field variants, and cell culture-attenuated GII strains, was shown to reduce pathogenicity but compromise immunogenicity. These results, which link S protein glycosylation to viral attenuation during both natural field transmission and laboratory cell passaging with poor cross-genogroup protection, provide a molecular basis for improving vaccine design.

## RESULTS

### Analysis of the variation pattern of the glycosylation site at position 381 in the S protein of PEDV during field epidemics and cell passaging and verification of its glycosylation

**Fig 1:**
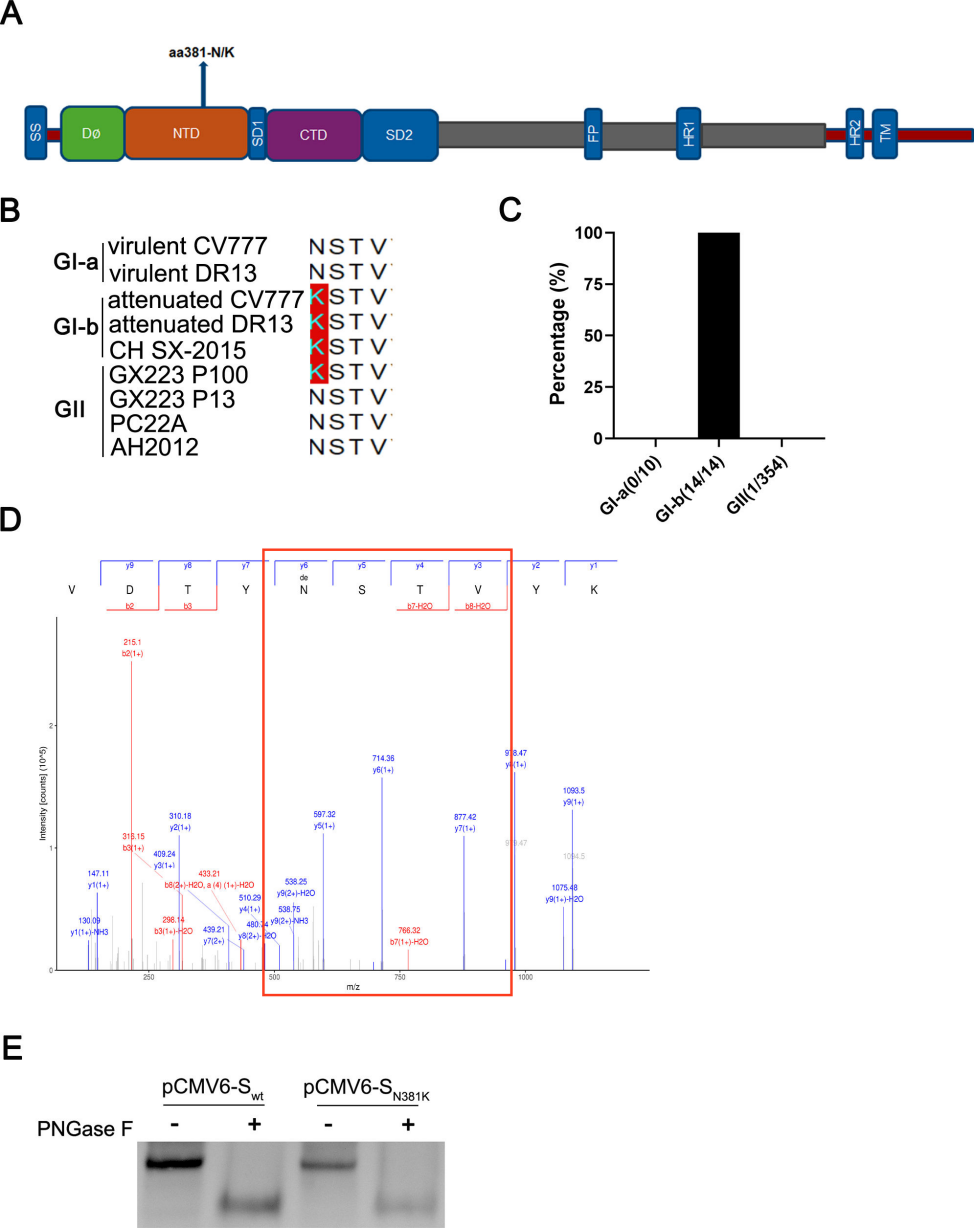
Analysis of the variation pattern of the glycosylation site at position 381 in the S protein of PEDV during field epidemics and cell passaging and verification of its glycosylation. (**A**) Schematic diagram of the location of the N381 glycosylation site in the PEDV S protein. (**B–C**) Variation characteristics of the glycosylation site at position 381 during epidemic spread and cell passaging. (**D**) Verification of the glycosylation site at position 381 by mass spectrometry. (**E**) Western blotting verification of the glycosylation site at position 381.

Glycosylation modification of viral proteins plays crucial roles in viral infection and pathogenesis (20). To study the mechanisms of PEDV virulence variation during both natural epidemics and cell culture serial passaging, we focused on the glycosylation patterns of the PEDV S protein. Sequence alignment of diverse PEDV strains revealed that the predicted glycosylation site mutation (N381K) in the S protein NTD predominantly occurs in low-virulence GI-b strains but not in highly virulent GII strains (Fig. 1A through C). Viruses typically undergo attenuation during high-passage serial propagation, a characteristic also shared by cell culture-attenuated GI vaccine strains. Surprisingly, an identical glycosylation mutation emerged in the 100th passage (P100) of the GII strain GX223 (Fig. 1B), which was a particularly unexpected finding given the stochastic nature of viral mutations. These observations collectively suggest that the N381K mutation in the S protein NTD may play a regulatory role in viral pathogenicity. Mass spectrometry and Western blotting analyses confirmed that the asparagine residue at position 381 (N381) is indeed glycosylated (Fig. 1D and E). These results demonstrate that the N381 glycosylation mutation (N381K) in the PEDV S protein likely contributes to variations in virulence during both natural transmission and laboratory cell passaging.

### Pathogenicity evaluation of low-passage and high-passage generations of strain GX223

**Fig 2:**
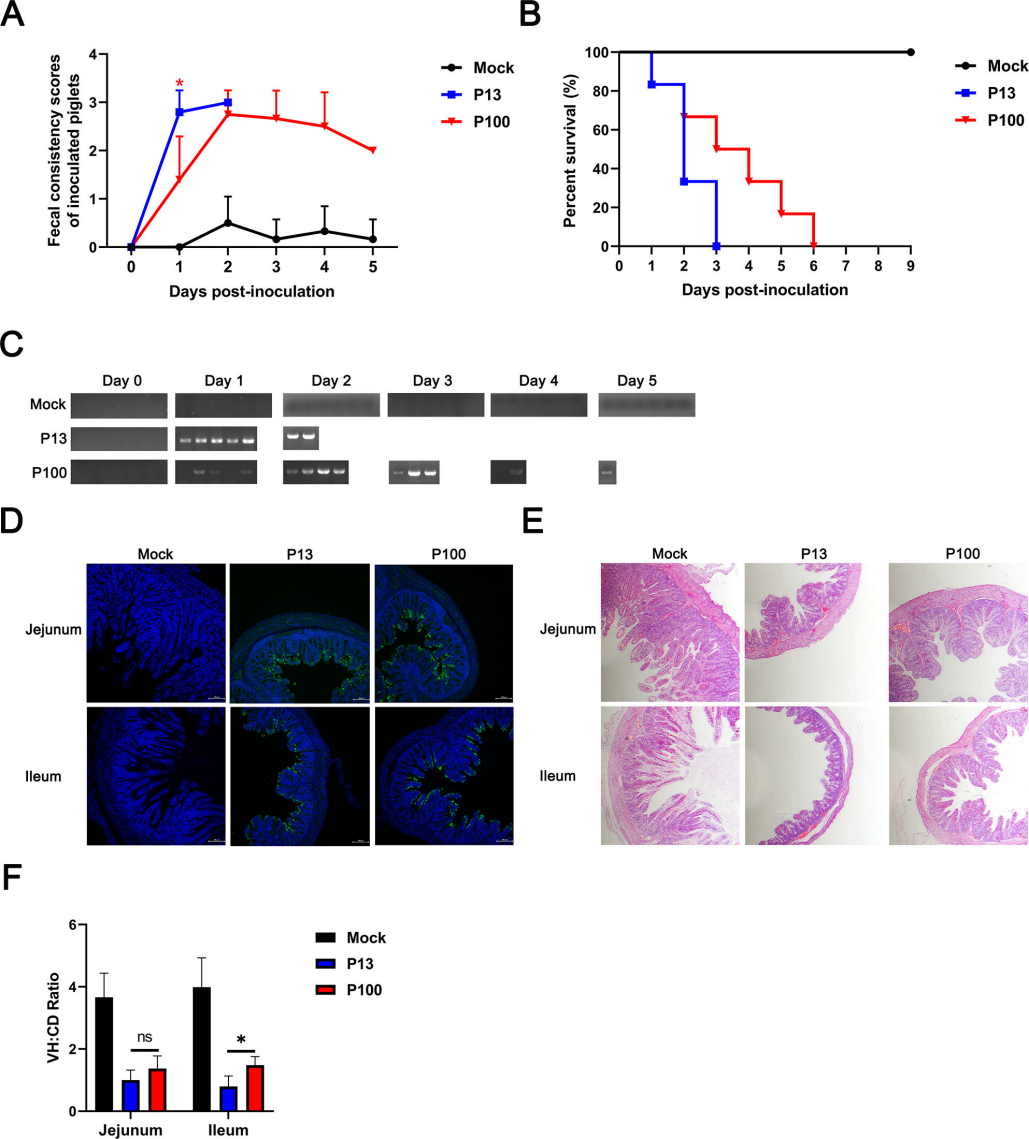
Pathogenicity evaluation of GX223 strains at different passages. (**A**) Fecal scores of the pigs. Fecal scores were scored as follows: 0, solid; 1, pasty; 2, semiliquid; and 3, liquid. Each line indicates the mean score of a group. (**B**) Survival curves of the piglets. (**C**) Fecal shedding of PEDV RNA. Viral RNA was isolated from rectal swab samples daily and subjected to RT-PCR to determine the degree of fecal shedding of PEDV RNA. (**D**) IHC staining of PEDV N proteins in the jejunum and ileum. (**E**) Hematoxylin and eosin (H&E) staining of the jejunum and ileum from dying or euthanized piglets. (**F**) The villous height/crypt depth (VH:CD) ratios of piglets. Ten villi from each intestinal section were measured. The error bars indicate the standard deviations. The significance level is expressed as *P < 0.05.

To investigate potential changes in virulence following serial cell passaging, we conducted a pathogenicity assessment of strain GX223 at passage 13 (P13) and passage 100 (P100). Compared with P13-infected piglets, piglets challenged with P100 (N381K) presented markedly attenuated clinical symptoms, including milder diarrhea (a significant difference in fecal scores was observed on the first day), delayed time to death, and delayed fecal virus shedding (Fig. 2A through C). Immunohistochemical (IHC) staining showed extensive and comparable viral antigen distribution in the small intestines of both P13 and P100 groups (Fig. 2D). Histopathological analysis confirmed that compared with P13 infection, P100 infection induced milder intestinal lesions (Fig. 2E and F). These results collectively demonstrate that cell culture passage leads to attenuation of strain GX223. Consistent with our initial hypothesis, the S protein N381K mutation appears to contribute to partial attenuation of high-passage generation in strain GX223.

### Rescue of a recombinant strain with the N381K mutation in the PEDV S protein and characterization of the rPEDV-S_N381K_

**Fig 3:**
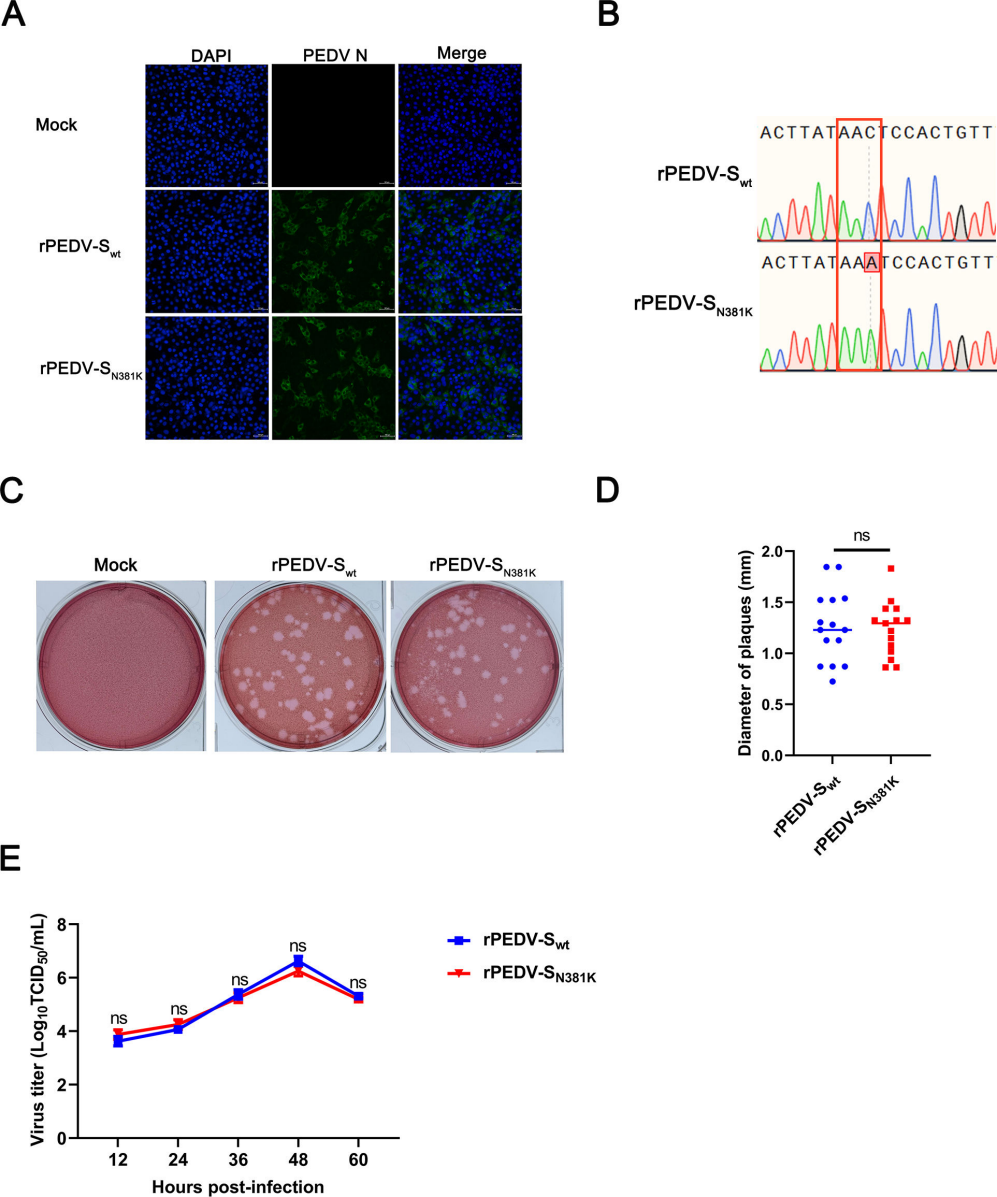
*In vitro* characterization of the recombinant strains. (**A**) VERO cells were infected with rPEDV-S_wt_ and rPEDV-S_N381K_. Infected cells were fixed at 24 hpi and immunolabeled with Fluorescein (FITC)-AffiniPure Goat Anti-Mouse IgG (H+L). Nuclei were labeled with 4-6-diamidino-2-phenylindole (DAPI) (blue). (**B**) VERO cells were infected with rPEDV-S_wt_ and rPEDV-S_N381K_. Infected cells were harvested at 36 hpi. Total RNA was extracted and reverse-transcribed. The rescue of the rPEDV-S_wt_ and rPEDV-S_N381K_ strains was subsequently identified by sequencing. (**C**) A plaque formation assay was performed. (**D**) The size of the plaques was calculated using ImageJ software. (**E**) VERO cells in 12-well plates were infected with the rPEDV-S_wt_ and rPEDV-S_N381K_ strains at an MOI of 0.1. The supernatant was harvested at 12, 24, 36, 48, and 60 hpi and titrated on VERO cells.

A DNA-launched reverse genetics system has been successfully developed by our group (21). To explore the effect of the N381K mutation in the PEDV S on the viral behaviors, a recombinant strain rPEDV-S_N381K_ was constructed using the rPEDV-S_wt_ strain as the backbone. Immunofluorescence assay (IFA) (Fig. 3A) and Sanger sequencing (Fig. 3B) indicated that rPEDV-S_N381K_ was successfully rescued. Compared with the parental rPEDV-S_wt_ strain, the rPEDV-S_N381K_ strain formed plaques of comparable size and exhibited similar growth kinetics (Fig. 3C through E). These results indicated that a recombinant strain with the N381K mutation in the PEDV S protein was obtained and that the recombinant strain exhibited similar growth kinetics in comparison with the parent strain *in vitro*.

### The rPEDV-S_N381K_ strain was partially attenuated in 2-day-old piglets

**Fig 4:**
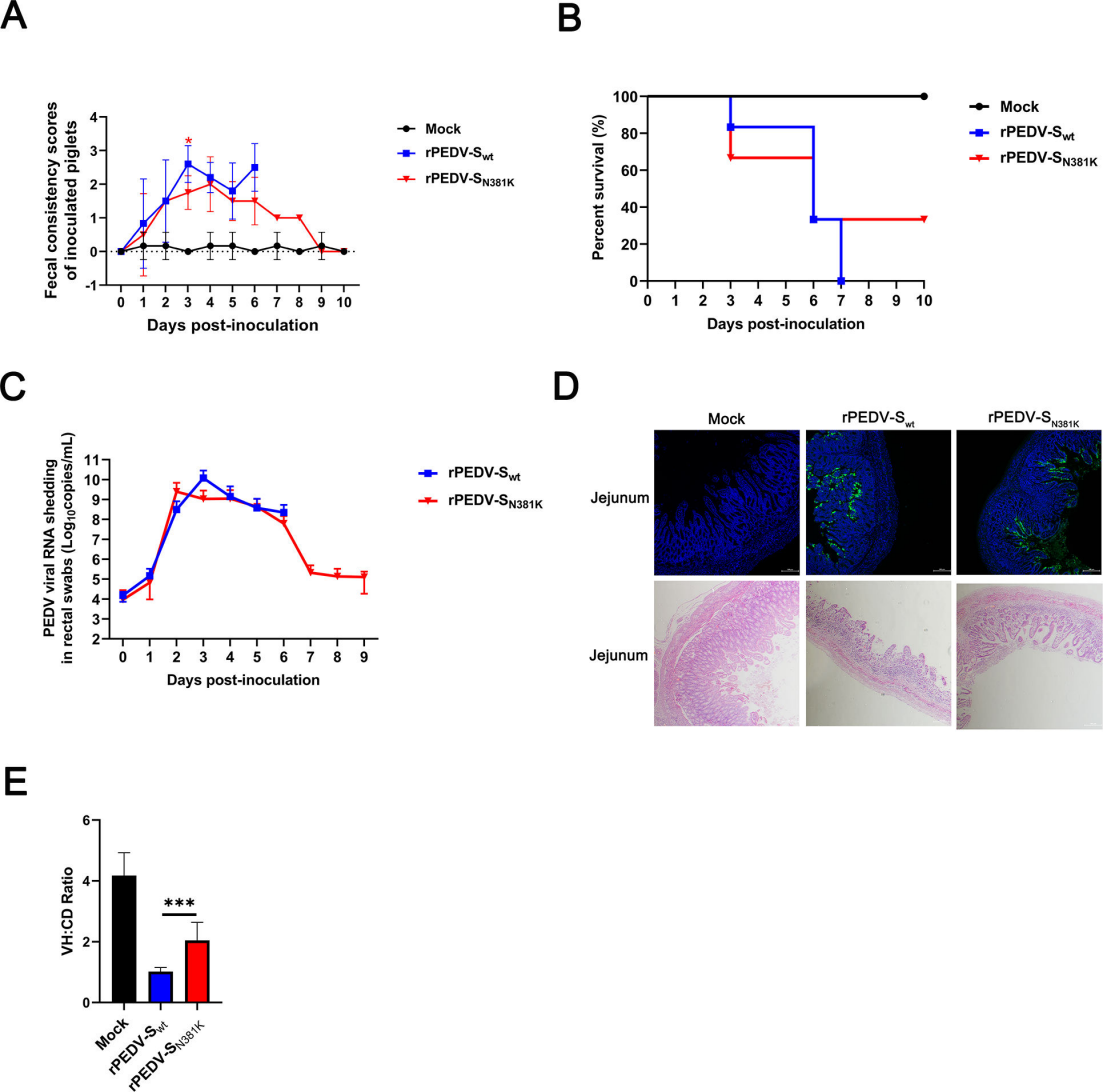
Pathogenicity of the rPEDV-S_N381K_ strain in 2-day-old piglets. (**A**) Fecal scores of pigs. Fecal scores were scored as follows: 0, solid; 1, pasty; 2, semiliquid; and 3, liquid. Each line indicates the mean score of a group. (**B**) Survival curves of piglets. (**C**) Fecal shedding of PEDV RNA. Viral RNA was isolated from rectal swab samples daily and subjected to RT-qPCR to determine the PEDV N gene RNA copies. (**D**) IHC staining and H&E staining of the jejunum from dying or euthanized piglets. (**E**) VH:CD ratios of piglets. Ten villi from each intestinal section were measured. Error bars indicate standard deviations. The significance level was expressed as *** *P* < 0.001.

To assess the pathogenesis of rPEDV-S_N381K_, eighteen 2-day-old piglets were randomly divided into three groups, with six pigs in each group. The pigs were orally inoculated with the rPEDV-S_wt_ and rPEDV-S_N381K_ viruses at a dose of 10^5^ TCID_50_ or mock infected with Dulbecco’s modified Eagle medium (DMEM). rPEDV-S_N381K_-inoculated pigs showed a lower mean diarrhea score than rPEDV-S_wt_-inoculated pigs from Day 3 post-infection, with a significant difference observed on Day 3 (Fig. 4A). However, diarrhea scores showed no significant differences on the other days, accompanied by large error bars, which may reflect the limitation of small animal numbers. Besides, two pigs (2/6) in the rPEDV-S_N381K_ group survived until the endpoint, whereas all animals (0/6) in the rPEDV-S_wt_ group died (Fig. 4B). Compared to the rPEDV-S_wt_-inoculated group, piglets in the group rPEDV-S_N381K_ showed comparable viral fecal RNA shedding (Fig. 4C). IHC staining showed extensive and comparable viral antigen distribution in the small intestines of both rPEDV-S_wt_ and rPEDV-S_N381K_ groups (Fig. 4D). H&E) staining revealed that the rPEDV-S_N381K_ strain caused milder histopathological lesions to intestinal villi compared to the rPEDV-S_wt_-inoculated group, but more serious intestinal villi damage than the mock group (Fig. 4D and E). These data suggested that the rPEDV-S_N381K_ strain was partially attenuated in 2-day-old piglets.

### The rPEDV-S_N381K_ strain was partially attenuated in 5-day-old piglets

**Fig 5:**
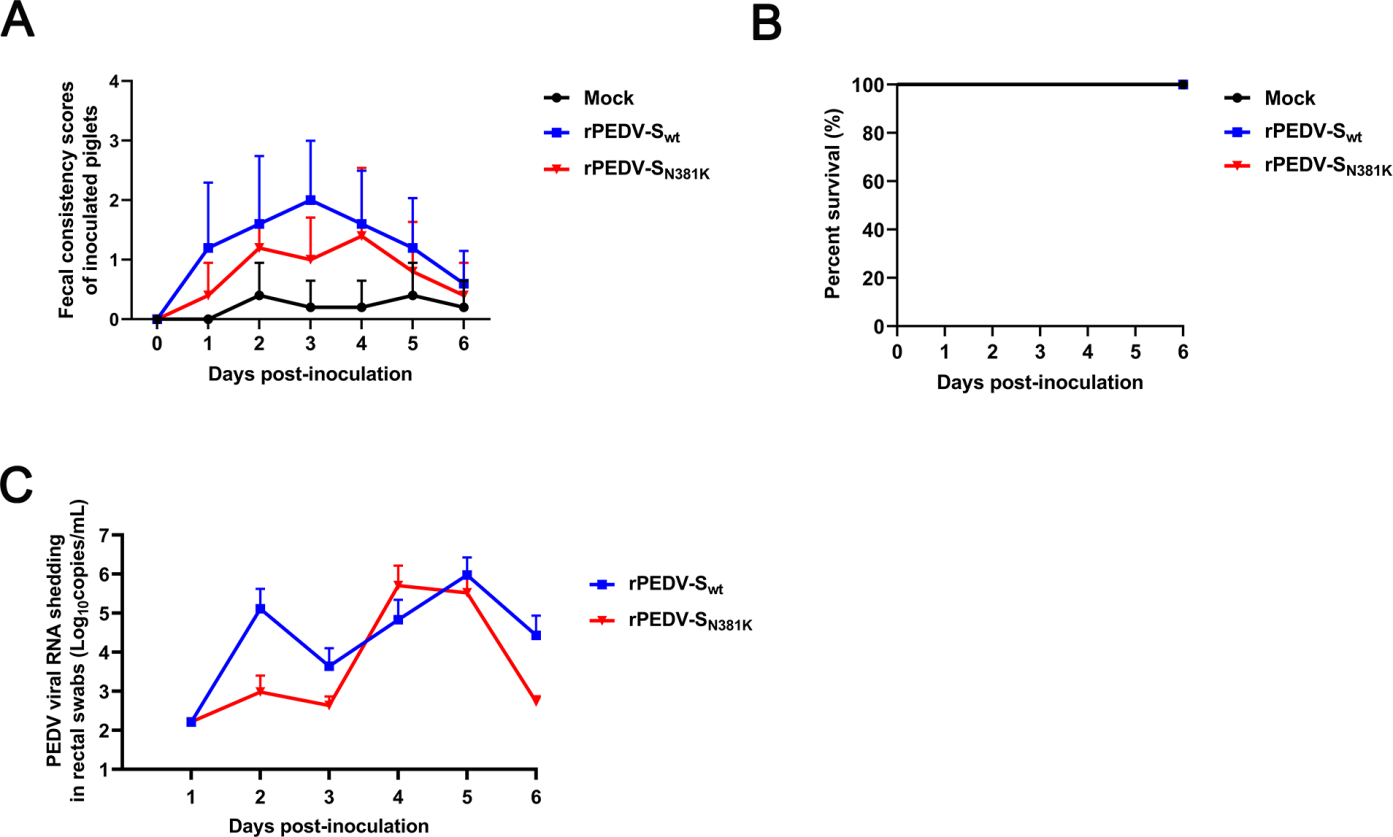
Pathogenicity of the rPEDV-S_N381K_ strain in 5-day-old piglets. (**A**) Fecal scores of pigs. Fecal scores were scored as follows: 0, solid; 1, pasty; 2, semiliquid; and 3, liquid. Each line indicates the mean score of a group. (**B**) Survival curves of piglets. (**C**) Fecal shedding of PEDV RNA. Viral RNA was isolated from rectal swab samples daily and subjected to RT-qPCR to determine the PEDV N gene RNA copies.

To further assess the pathogenesis of rPEDV-S_N381K_, fifteen 5-day-old piglets were randomly divided into three groups, with five pigs in each group. The pigs were orally inoculated with the rPEDV-S_wt_ and rPEDV-S_N381K_ viruses at a dose of 10^4.5^ TCID_50_ or mock infected with DMEM. Compared to the rPEDV-S_wt_ groups, although there was no significant difference in fecal scores, piglets in the rPEDV-S_N381K_ group exhibited a lower mean diarrhea score (Fig. 5A)**,** while no pigs died in either group (Fig. 5B). Additionally, piglets in the rPEDV-S_N381K_ group exhibited lower mean fecal viral loads than those in the rPEDV-S_wt_ group on all days except Day 4, which suggests a delayed onset of shedding in the former group (Fig. 5C). These results suggested that the rPEDV-S_N381K_ strain was partially attenuated in 5-day-old piglets. However, a limitation of these results must be considered. The high variability in the data, as shown by the large error bars, prevented statistical analysis, most likely due to the limited animal numbers.

### The rPEDV-S_N381K_ strain elicited suboptimal protective immunity against the parental strain

**Fig 6:**
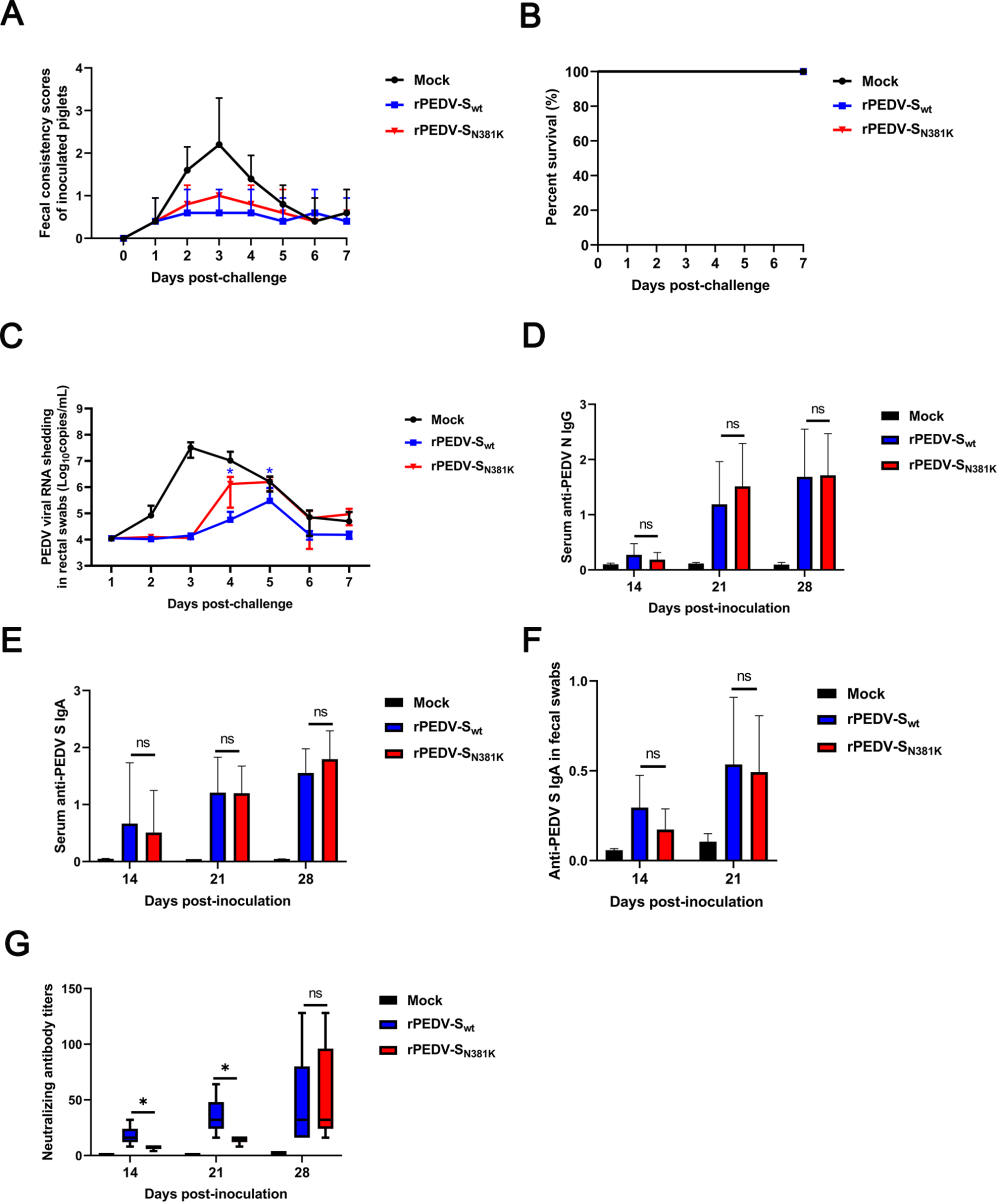
Protection induced by the rPEDV-S_wt_ and rPEDV-S_N381K_ strains in pigs. (**A**) Fecal scores of pigs post-challenge. Fecal scores were scored as follows: 0, solid; 1, pasty; 2, semiliquid; and 3, liquid. Each line indicates the mean score of a group. (**B**) Survival curves of piglets post-challenge. (**C**) Evaluation of fecal PEDV RNA shedding in pigs post-challenge. Viral RNA was isolated from rectal swab samples daily and subjected to RT-qPCR to determine the PEDV N gene RNA copies. (**D**) The levels of anti-PEDV N IgG in sera collected at 14 dpi, 21 dpi, and 28 dpi. (**E**) The levels of anti-PEDV S IgA in sera collected at 14 dpi, 21 dpi, and 28 dpi. (**F**) The levels of anti-PEDV S IgA in feces collected at 14 dpi and 21 dpi. (**G**) Virus-neutralizing antibody titers in sera collected at 14 dpi, 21 dpi, and 28 dpi. Error bars indicate standard deviations. The significance level was expressed as * *P* < 0.05.

At 21 dpi, all the pigs were challenged with the virulent rPEDV-S_wt_ at a high dose of 10^6^ TCID_50_/pig. Pigs in the mock group developed severe diarrhea, whereas no diarrheal symptoms or statistically significant differences in fecal scores were observed between groups rPEDV-S_N381K_ and rPEDV-S_wt_ (Fig. 6A). No pigs in the three groups died (Fig. 6B). Unexpectedly, while pigs in both the rPEDV-S_N381K_ and rPEDV-S_wt_ groups exhibited reduced fecal viral shedding compared to the mock group, pigs in the rPEDV-S_N381K_ group showed higher mean fecal viral shedding versus the rPEDV-S_wt_ group, with a statistically significant difference emerging on Days 4 and 5 (Fig. 6C). This observation correlated with the findings: although comparable levels of serum IgG, serum IgA, and fecal IgA were detected in both groups (Fig. 6D through F), the rPEDV-S_N381K_ group showed significantly lower neutralizing antibody titers against the parental strain at 14 and 21 days post-infection (Fig. 6G). However, at the 21 days post-infection, the immune memory cells in the piglets of both groups may be comparable, and there is no difference in the neutralizing antibody levels between the two groups after the parental virus challenge (Fig. 6G). In summary, these data indicate that rPEDV-S_N381K_ reduced protective immunity against the challenge of virulent rPEDV-S_wt_.

### The N381K mutant in the S protein may alter intermolecular interactions between amino acids within antigenic epitopes

**Fig 7:**
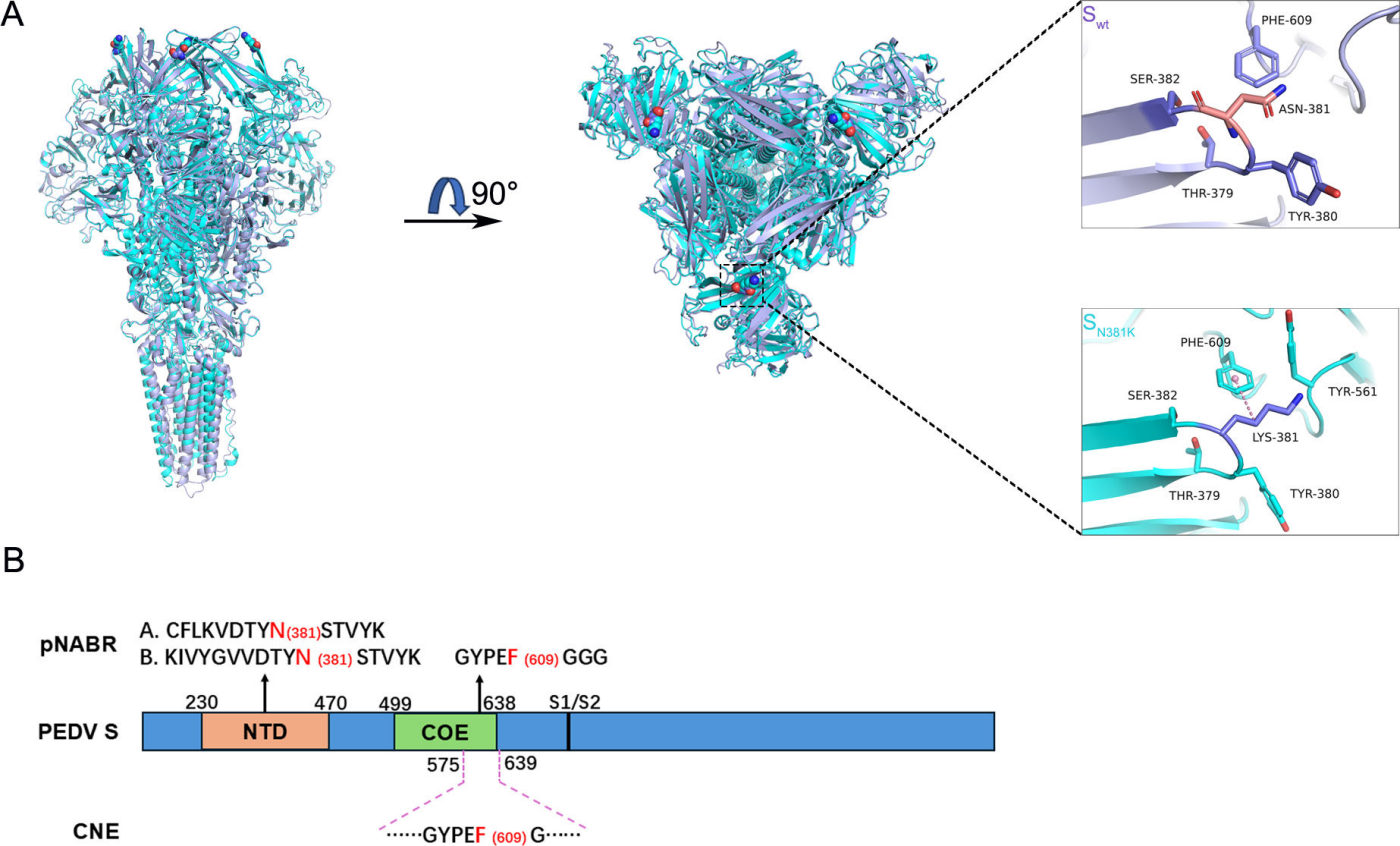
Structural prediction of S protein of the rPEDV-S_wt_ and rPEDV-S_N381K_ strains. (**A**) The three-dimensional conformation of the recombinant spike protein was modeled employing AlphaFold 3. (**B**) Schematic diagram of several neutralizing epitopes or potential neutralizing epitopes on the PEDV S protein.

To analyze the molecular mechanism by which the N381K mutant attenuated virulence and reduced immunogenicity, trimeric S proteins were built. Based on the modeling and the experimental results above, we hypothesized the mechanism by which the N381K mutation affects viral virulence and immunogenicity. As shown in Fig. 7A, the polar but uncharged asparagine 381 of the S_wt_ protein fails to form polar interactions with surrounding residues. Upon mutation to lysine, the positively charged side chain orients its hydrophobic aliphatic carbons to engage in interactions with the aromatic ring of phenylalanine 609 (F609). The F609 is located in the CO-26K equivalent region (COE), a neutralizing epitope widely used in the development of PEDV subunit vaccines (22, 23). Furthermore, the C-terminal segment of COE (amino acids 575–639) was identified as a conformational neutralizing epitope (CNE) containing F609 (24) (Fig. 7B). Moreover, synthetic peptides corresponding to the predicted epitopes (“CFLKVDTYNSTVYK,” “KIVYGVVDTYNSTVYK,” and “GYPEFGGG”) were shown to inhibit the neutralizing activity of PEDV-positive sera, suggesting that these predicted antigenic sites were likely potential neutralizing antibody binding regions (pNABR) (25). N381 is located within epitopes “CFLKVDTYNSTVYK” and “KIVYGVVDTYNSTVYK,” while F609 resides in epitope “GYPEFGGG” (Fig. 7B). Collectively, both N381 and F609 are situated within putative neutralizing epitopes or CNEs. The N381 mutation itself, or its altered interactions with neighboring residues in these antigenic epitopes, may influence viral immunogenicity and other characteristics.

## DISCUSSION

Coronaviruses, as single-stranded positive-sense RNA viruses, are prone to genomic mutations during natural epidemics, which may alter their virulence and immunogenicity. For example, the T492I mutation in nsp4 of SARS-CoV-2 was shown to increase viral replication and transmissibility while improving immune evasion (7), and the R203M and D377Y mutations in the N protein of SARS-CoV-2 promoted viral replication and infectivity by inhibiting interferon production (26). The sudden emergence of virulent PEDV variants (GII strains) in 2010 caused devastating losses to the global swine industry, yet the mechanisms of PEDV virulence variation remain poorly understood (27). Through comprehensive sequence analysis, we revealed that during the evolution from low-virulence, less epidemic GI-b strains to high-virulence, highly epidemic GII strains, a novel glycosylation site emerged at position 381 of the spike protein (from KSTV to NSTV) (Fig. 1) and that the strain carrying the N381K mutation exhibits reduced virulence (Fig. 4). This study demonstrates that the glycosylation site at position 381 (N381) in the PEDV S protein partially contributed to the increased viral pathogenicity observed in epidemic strains since 2010.

LAVs represent a crucial strategy for controlling disease. The development of PEDV LAVs, such as the classical CV777 and DR13 strains, is typically achieved through serial passaging in VERO cells, during which the viral genome accumulates numerous mutations accompanied by reduced virulence and sometimes altered immunogenicity (28), a phenomenon also observed in our study (Fig. 2). The findings of this study are entirely consistent with the observed limitations of classical vaccines against circulating field strains, wherein highly virulent parental strains (e.g., pathogenic CV777 and DR13) are attenuated through serial passaging to develop vaccine strains that often demonstrate poor protection against prevalent epidemic variants (29). The unexpected discovery suggests that the N381K mutation in the PEDV spike protein may contribute to a reduction in the cross-protective efficacy of the vaccine.

Posttranslational modifications, particularly glycosylation, play pivotal roles in regulating viral virulence, replication efficiency, and tissue tropism (30, 31). For example, Li et al. revealed that evolutionary glycosylation mutations in the S protein of SARS-CoV-2 significantly impact antigenicity, neutralization, and S protein stability (19). However, these studies focused primarily on how mutations in S protein glycosylation sites during field epidemics affect viral infectivity, virulence, and neutralization susceptibility. Our study provides comprehensive evidence that N381K glycosylation site mutation contributes to variations in virulence and immunogenicity among circulating field strains and passages in nonporcine cell cultures (Fig. 4 and 6). These multifaceted findings establish the N381 site as an evolutionary hotspot of PEDV.

The NTD of the coronavirus S protein likely has conserved functions in modulating viral evolution (30). Mutations in the NTD of SARS-CoV-2 are correlated with immune evasion and viral fitness (32). For example, ΔH69/V70 in the SARS-CoV-2 S NTD region regulates viral infectivity (33), and ΔY144 or K147E+W152R in the SARS-CoV-2 S NTD region is related to neutralizing antibody evasion (34, 35). The N381 glycosylation site is also located in the NTD region of the PEDV S protein (Fig. 1). We found that N381 had limited impact on viral replication, which is consistent with the findings of previous reports (36). However, N381 is associated with immune evasion and virulence evolution (Fig. 4 and 6). These results indicated that the modulation of coronavirus evolution by the NTD region of the S protein is likely conserved.

In summary, the glycosylation site mutation at position 381 (N381) in the PEDV S protein contributes to driving virulence evolution during both natural field transmission and cell culture adaptation while simultaneously affecting cross-protective efficacy among heterologous strains. Our findings significantly advance the understanding of the molecular mechanisms underlying PEDV virulence evolution and immune evasion and provide new insights for the rational design of PEDV LAVs.

Despite our findings, this study has several limitations. First, the sample sizes were relatively small (*n* = 5–6 piglets per group). The considerable variability in the dispersion observed in some of our data sets, although common in biological studies, further reduces the ability to detect statistically significant differences with confidence. Second, we propose a mechanism by which the N381 mutation alters interactions with neighboring residues in these antigenic epitopes, which may influence viral immunogenicity and other characteristics. Our hypothesis is primarily derived from bioinformatics predictions that suggest that the N381 mutation occurs in a key functional domain. While this is a plausible mechanism that aligns with our phenotypic observations, it is critical to emphasize that this mechanistic explanation remains highly speculative.

## MATERIALS AND METHODS

### Cells and viruses

VERO cells were preserved in our laboratory (37). VERO cells were cultured in DMEM (Gibco, CA) supplemented with 10% fetal bovine serum ( TransGen Biotech, China), 100 U/mL penicillin, and 100 µg/mL streptomycin. Previously, our group constructed and successfully rescued the recombinant strain rCH/SX/2016-S_HNXP_ (rPEDV-S_wt_) (21). The GX223 strain (GenBank accession number: PV982377) was isolated and preserved by our group. The propagation of PEDV was performed in VERO cells.

### Western blotting

HEK-293T cells seeded in six-well plates were transfected with plasmids expressing full-length S proteins (pCMV6-S_wt_ and pCMV6-S_N381K_). At 36 h after transfection, the cells were lysed with 200 µL of ice-cold RIPA buffer for 30 min on ice, and then, the proteins in the supernatant were collected after centrifugation 12,000 × *g* at 4°C. The detection of N381 glycosylation using Western blotting was performed as previously reported (31). The samples were separated on a 7% Tris-acetate gel (P0534S, Beyotime) using the BeyoGel Tris-acetate SDS running buffer (P0749, Beyotime) and transferred onto PVDF membranes using western transfer buffer (P0021B, Beyotime). The membranes were blocked with 5% nonfat milk in PBST for 2 h at room temperature and then incubated with the PEDV S monoclonal antibody at 4°C overnight. The membranes were washed with PBST and then incubated with goat anti-mouse IgG (H+L) HRP-conjugated secondary antibody (31430, Thermo Fisher Scientific) for 1 h at room temperature. After washing, the target proteins were detected with the enhanced WesterBright ECL Kit (K-12045-D50, Advansta).

### Strategies for constructing and rescuing chimeric full-length cDNA clones of PEDV

The rPEDV-S_N381K_ strain was generated using a similar strategy as before with slight modification (21). In brief, VERO cells were grown to 80% confluency in a six-well plate, and 2.5 µg of the recombinant BAC plasmids was transfected into VERO cells using the Lipofectamine 3000 transfection reagent. The CPE was monitored daily after transfection. When CPE was obvious, the cells and supernatants were collected and freeze-thawed for propagation.

### Sequencing analysis identified the N381K mutation of the PEDV S protein

The N381K mutation of the PEDV S protein was analyzed by sequencing. VERO cells were infected with rPEDV-S_wt_ and rPEDV-S_N381K_ and collected at 36 hpi. The RNA of the samples was extracted using TRIzol reagent (TaKaRa, Japan) and reverse transcribed using HiScript II Q RT SuperMix for qPCR (Vazyme, China) according to the manufacturer’s instructions. For sequencing, the fragments were amplified using the primers PEDV-S-F and PEDV-S-R. The sequences of the primers used are listed in Table 1.

**TABLE 1:** Primers in this study

Primer name	Sequence (5′−3′)	Usage	Ref.
PEDV-S-F PEDV-S-R	AGCCTACCACAAGATGTCAC CTAGTGTCAACACAGAAAGAAC	Sequencing	
PEDV-M-F	TTCGGTTCTATTCCCGTTGATG	RT-PCR	
PEDV-M-R	CCCATGAAGCACTTTCTAACTATC		
PEDV-N-F	GAATTCCCAAGGGCGAAAAT	RT-qPCR	(38)
N gene probe	FAM-CGTAGCAGCTTGCTTCGGACCCA-BHQ		
PEDV-N-R	TTTTCGACAAATTCCGCATCT		

### Indirect IFA

IFA was performed as before with slight modification (21). VERO cells were infected with rPEDV-S_wt_, rPEDV-S_N381K_, or mock. Cells were washed with phosphate-buffered saline (PBS) and fixed with 4% paraformaldehyde for 10 min at 37°C, followed by membrane permeabilization with 0.25% Triton X-100 in PBS for 10 min at 37°C. Cells were blocked with 1% BSA at 37°C for 30 min and then incubated with mouse anti-N polyclonal antibody at a dilution of 1:1,000 at 37°C for 1 h. Cells were washed with PBS three times and incubated with Fluorescein (FITC)-AffiniPure Goat Anti-Mouse IgG (H+L) at 1:200 at 37℃ for 1 h. Cells were washed with PBS three times and stained with DAPI for 10 min at room temperature. The images were captured with a fluorescence microscope.

### Growth kinetics

Multistep growth kinetics were calculated as before with slight modifications (21). VERO cells in 12-well plates were infected with PEDV at an MOI of 0.1. After 1 h of absorption, the cells were washed with PBS three times and maintained in maintenance medium. The virus titers of the supernatants at the indicated time points were determined by the TCID_50_ assay.

### Plaque assay

The plaque assay was performed according to a previously described method (21). VERO cells in six-well plates were infected with 2 mL of 10-fold serially diluted PEDV. After 1 h of absorption, the cells were washed with PBS three times and then overlaid with 1% low-melting agarose. Plaques were visualized with neutral red dye.

### Evaluation of the pathogenicity of GX223

Eighteen piglets, which were negative for transmissible gastroenteritis virus (TGEV), PEDV, porcine delta coronavirus (PDCoV), and rotavirus (RV), were randomly divided into three groups, with six pigs in each group. At 2 days of age, piglets were orally inoculated with a dose of 10^4^ TCID_50_/pig of the 13th and 100th passages of strain GX223 or with DMEM. Daily clinical observations were performed on all the piglets, with necropsy examinations conducted following any mortality events. Rectal swabs were collected daily, and viral RNA shedding from the rectal swabs was determined by RT-PCR. The rectal swabs were thoroughly mixed with 1 mL of DMEM. After centrifugation at 13,000 rpm at 4°C for 10 min, the supernatants were collected for subsequent analysis. RNA was extracted from equal volumes of the supernatant using RNAiso Plus (TaKaRa, Japan). Afterward, reverse transcription was performed using HiScript II Q RT SuperMix for qPCR (Vazyme, China). Equal volumes of cDNA were used to perform the RT-PCR assays with the primers PEDV-M-F and PEDV-M-R, as shown in Table 1.

### Evaluation of the pathogenicity and immunogenicity of the rPEDV-S_N381K_ strain

Eighteen 2-day-old piglets that were negative for TGEV, PEDV, PDCoV, and RV were randomly divided into three groups, with six pigs in each group. The challenge group piglets were orally inoculated with parental or recombinant PEDV at 10^5^ TCID_50_/pig. The negative control group was inoculated with an equal volume of DMEM. Rectal swabs were collected daily, and viral RNA shedding copies in the rectal swab samples were determined by the AceQ Universal U+ Probe Master Mix V2 reagent (Vazyme, China) using the primers PEDV-N-F, PEDV-N-R, and the N gene probe, as shown in Table 1. The severity of diarrhea was scored as follows: 0, solid; 1, pasty; 2, semiliquid (mild diarrhea); and 3, liquid (severe diarrhea) (39). Necropsy examinations were conducted following any mortality events. The tissues were fixed in a 4% paraformaldehyde solution and stained with H&E. IHC staining was also performed using an antibody against the PEDV N protein as the primary antibody.

Fifteen 5-day-old piglets that were negative for TGEV, PEDV, PDCoV, and RV were randomly divided into three groups, with five pigs in each group. The challenge group piglets were orally inoculated with parental and recombinant PEDV at 10^4.5^ TCID_50_/pig. The negative control group was inoculated with an equal volume of DMEM. All the piglets were subjected to daily clinical observations. Rectal swabs were collected daily. The severity of diarrhea was scored, and the number of viral RNA shedding copies in the rectal swab samples was determined as described above. At 21 dpi, the surviving pigs were challenged orally with 10^6^ TCID_50_ of rPEDV-S_wt_. Clinical signs and mortality were monitored every day. Rectal swabs were collected daily. The severity of diarrhea was scored, and the number of viral RNA shedding copies in the rectal swab samples was determined as described above.

### ELISA for anti-PEDV N IgG and S IgA detection

At 14, 21, and 28 dpi, blood samples were collected and stored at 4℃ overnight. Afterward, the samples were centrifuged at 3,000 × *g* for 10 min, after which the serum samples in the supernatants were collected. Serum anti-PEDV N IgG was detected by the ID Screen Porcine Epidemic Diarrhea Virus Antibody Detection Elisa Kit (IDvet, France), and anti-PEDV S IgA in the serum and feces was detected by the PEDV IgA Antibody Test Kit (IDEXX, USA) according to the manufacturer’s instructions.

### Virus neutralization test

The virus neutralization test for the detection of PEDV-neutralizing antibodies was performed as described previously (40), with some modifications. Briefly, heat-inactivated sera (56°C for 30 min) were serially diluted twofold with DMEM. Afterward, the serum samples were mixed with equal volumes of virus solution containing 200 TCID_50_/0.1 mL of PEDV. Following incubation at 37°C for 90 min, the mixture was added to VERO cells in 96-well plates. After incubation at 37°C for 1 h, the inoculum was discarded. Afterward, the cells were washed with PBS three times and cultured with DMEM. Intracellular CPE was observed for 4–5 days.

### Prediction of protein structures

The N-glycosylation sites were predicted using the NetNGlyc 1.0 server available at https://services.healthtech.dtu.dk/services/NetNGlyc-1.0/. The sequences of PEDV S_wt_ and S_N381K_ were submitted to the AlphaFold 3 server, and the default parameters were used for the S protein trimer structure. Structural analysis and visualization were performed using PyMOL 2.3.0.

### Statistical analysis

The statistical analyses were performed using GraphPad Prism 8.0.2. The data were analyzed by an unpaired *t*-test or one-way analysis of variance. *P < 0.05, **P < 0.01, and ***P < 0.001 were considered to indicate statistical significance. The error bars indicate the means ± standard deviations (SDs).

## Data Availability

All data generated during the current study are included in the article.
